# Bypassing Androgens: Retina-Targeted 17β-Estradiol Delivery via DHED Eye Drops Ameliorates Orchiectomy-Induced Shifts in the Male Rat Visual Cortex Proteome

**DOI:** 10.3390/antiox15080976

**Published:** 2026-08-06

**Authors:** Khadiza Zaman, Ammar Kapic, Vien Nguyen, Laszlo Prokai, Katalin Prokai-Tatrai

**Affiliations:** Department of Pharmacology and Neuroscience, University of North Texas Health Science Center, Fort Worth, TX 76107, USA; khadiza.zaman@unthsc.edu (K.Z.); ammarkapic@my.unthsc.edu (A.K.); vien.nguyen@unthsc.edu (V.N.); laszlo.prokai@unthsc.edu (L.P.)

**Keywords:** androgen deprivation, bioinformatics, DHED, Ingenuity Pathway Analysis^®^, neuroproteomics, mass spectrometry, orchiectomy, oxidative stress, proteomic dysregulation, male rat visual cortex

## Abstract

Mass spectrometry-based proteomics has become instrumental for elucidating the molecular mechanisms of neurodegeneration, enabling the identification of disease-associated proteomic alterations and protein-level responses to therapeutic interventions. Building on our previous proteomic characterization of the orchiectomized (ORX) Brown Norway male rat retina as a model of androgen deprivation, we extend this investigation to the visual cortex (VC), a key node in the visual pathway. Here we report the first comprehensive proteomic characterization of ORX-VC employing nanoflow liquid chromatography–tandem mass spectrometry complemented by bioinformatic pathway analyses and quantitative assessment of selected protein markers of ORX-induced changes. Our discovery-driven proteomics approach revealed over 500 differentially expressed proteins following ORX. Principal component analysis showed complete separation between ORX and naïve groups with no overlap, indicating a profound effect of androgen deprivation on the VC proteome. Ingenuity Pathway Analysis^®^ predicted that the widespread proteome dysregulation included oxidative stress as a primary contributor, alongside estrogen receptor (ESR) predominance over androgen receptor signaling, consistent with our prior findings in the ORX retina. To explore whether this ESR predominance translates to a benefit of E2 supplementation alone, independent of circulating testosterone, we utilized our DHED (10β,17β-dihydroxyestra-1,4-dien-3-one) prodrug as eye drops to target E2 formation into the retina. Quantitative assessment demonstrated that this approach resulted in attenuation of ORX-induced expression changes in a selected panel of key proteins. These findings warrant future studies to determine whether this effect extends across the full spectrum of ORX-induced proteomic shifts and confers a benefit for vision preservation.

## 1. Introduction

Neurodegeneration is a complex multifactorial process in both its initiation and propagation [1,2]. Although many clinically relevant contributing factors have been recognized over the years, our understanding of the underlying molecular mechanisms remains incomplete [3,4]. Recently, mass spectrometry (MS)-based proteomics has emerged as a valuable tool for uncovering the molecular underpinnings of neurodegenerative diseases, offering insights into proteome-wide alterations driven by condition-induced disruptions in proteostasis, as well as protein-level responses to treatments, thereby advancing both biomarker and drug discovery [5,6,7,8]. Our focus has been on ocular neurodegeneration—glaucoma in particular, where retinal ganglion cells (RGCs) represent the primary cellular target [9,10,11]. However, the retina is an extension of the central nervous system (CNS) and cannot be considered in isolation from the brain [12,13]. In normal visual function, the visual cortex (VC) receives most of its input from RGCs, whose axons form the optic nerve (ON), which crosses at the optic chiasm and relays signals to the VC via the lateral geniculate nucleus [14,15,16]. As a result, neurodegenerative signaling originating in the retina is not confined to the eye but can spread along the entire visual pathway. While neuroimaging studies have reported downstream VC changes in glaucoma [17,18,19], proteome-level consequences of this propagation at the level of the VC remain largely unexplored.

In our previous work, we characterized proteomic changes in the glaucomatous retina using preclinical animal models of the disease. Specifically, we evaluated the retinal proteomes of both male and female Brown Norway (BN) rats across two distinct injury mechanisms: a chronic, sustained elevation of intraocular pressure (IOP) and an acute mechanical insult via ON crush [9,10,11,20]. Beyond the injury itself, another critical factor in these animal models is gonadal status. Multiple lines of evidence indicate that sex hormones, particularly 17β-estradiol (E2), act as a potent pleiotropic neuroprotective agent [21,22,23,24]. While E2 is the dominant female sex hormone, it remains less clear in males whether testosterone (T) itself and/or its androgenic metabolites are neuroprotective, or whether this effect is instead attributable to E2, T’s metabolite through aromatase-catalyzed oxidation [25,26,27,28]. Regardless, the natural or iatrogenic loss of endogenous E2 in females has been generally associated with heightened susceptibility to neurodegenerative diseases affecting both the brain and the eye [29,30]. The effect of analogous androgen loss in males is, however, understudied.

To address this knowledge gap, we recently provided proteomics-based evidence, supported by bioinformatic analyses, of increased vulnerability to oxidative stress (OS) and, by extension, elevated neurodegenerative risk in the male BN rat retina following androgen deprivation due to orchiectomy (ORX) [31]. OS is one of the principal contributors to chronic neurodegeneration and arises from excessive production of and prolonged exposure to reactive oxygen and nitrogen species [32,33,34,35]. The CNS is highly sensitive to OS due to many factors, including high metabolic demand and abundant lipid content. In our study that compared ORX rats with age-matched intact counterparts [31], Ingenuity Pathway Analysis (IPA^®^) of differentially expressed proteins (DEPs) uncovered nearly 300 significantly regulated canonical pathways, pointing not only to ORX-induced OS but also mitochondrial dysfunction. Moreover, IPA^®^ predicted a predominance of estrogen receptor (ESR) signaling over androgen receptor (AR) signaling in the sex hormone-deprived retina despite the loss of T following ORX, suggesting that E2-mediated pathways potentially play a more prominent role in male retinal protection than previously recognized.

Given this framework, we next asked whether these effects extend to VC, a key component of the rat visual pathway. The present study therefore pursues two objectives. First, as no proteome characterization of the ORX-VC previously existed, we employed a discovery-driven proteomics approach to establish a foundational dataset and identify candidate pathways affected by androgen deprivation. IPA^®^-predicted pathway associations from this dataset, including OS and ESR-predominant signaling, formed the basis for our hypothesis that E2 supplementation alone, without reestablishing circulating T, would be sufficient to attenuate ORX-induced cortical proteome disruption. Second, we tested this hypothesis through quantitative assessment of a selected panel of ORX-impacted proteins using our DHED (10β,17β-dihydroxyestra-1,4-dien-3-one) prodrug [36], administered as eye drops to target E2 formation into the retina, without peripheral exposure to the hormone.

## 2. Materials and Methods

### 2.1. Chemicals and Reagents

DHED was prepared in our laboratory, as reported before [37,38]. Reagents were ordered from Millipore Sigma (St. Louis, MO, USA), except sequencing-grade trypsin (Promega, Madison, WI, USA) and Optima^®^ LC/MS-grade solvents, including water, formic acid (FA), and acetonitrile (ACN), which were obtained from Thermo Fisher Scientific (Waltham, MA, USA). Ketamine (100 mg/mL) and xylazine (20 mg/mL) used for euthanasia were purchased from Covetrus (Fort Worth, TX, USA).

### 2.2. Animals and Treatments

The ARVO declaration for the “Use of Animals in Ophthalmic and Vision Research” served as the basis for all animal-related procedures. Before the commencement of studies, all protocols were approved by our Institutional Animal Care and Use Committee (approval numbers: IACUC-2023-0012 approved on 2 May 2023 and renewed on 1 May 2026 as IACUC-2026-0007). Intact and ORX BN male rats (56–62 days old) were purchased from Charles River Laboratories (Wilmington, DE, USA). ORX surgery was performed by the supplier when the animals were 49–55 days old. The intact animals were considered the naive (reference) group. For euthanasia (exactly 28 days after receiving the animals), i.p. administration of ketamine (60 mg/kg body weight) and xylazine (10 mg/kg body weight) was performed. The brain was removed immediately, followed by a quick dissection to collect the VCs, which were then stored at −80 °C until processing for proteomics studies. For the present study, one VC (randomly selected as left or right) was used from each animal. In the first experimental setup, VCs from the naïve and ORX groups (*n* = 5, each) were used for the discovery-driven proteomic studies to survey the effect of ORX alone. In the second experimental design, ORX animals received bilaterally 10 μL of 0. 1% (*w*/*v*) DHED eye drops once daily (q. d., *n* = 5, designated as the DHED group) or the vehicle (20% *w*/*v* hydroxypropyl-β-cyclodextrin in saline, *n* = 5, designated as the Vehicle group) for 21 days, according to our previously used protocol [9,37]. Treatment started 6 days after receiving the animals from the supplier. Tissue collection was done 24 h after the last treatment.

### 2.3. Sample Preparation

VC tissues were suspended in 800 µL of 8 M urea dissolved in 25 mM ammonium bicarbonate (ABC) and homogenized for 30 s, with 3 repetitive cycles in a Bead Blaster^TM^ 24 refrigerated microtube homogenizer (Millipore Sigma, Rockville, MD, USA), followed by subsequent incubation for an hour at room temperature. The clear supernatant was collected by centrifugation; then, the protein content was estimated using a BioTek Synergy H1 microplate reader with a Take3 microplate (Agilent, Palo Alto, CA, USA) using two technical replicates, as reported before [31,37]. Samples were normalized to 100 µg of protein each and reduced with dithiothreitol and carbamidomethylated with 5 mM iodoacetamide at room temperature in the dark, according to standard protocols [31]. After diluting the samples with 25 mM ABC to reduce the urea concentration to <2 M, proteins were digested overnight at 37 °C with 2 μg trypsin per sample. Digestion was quenched by adding 5 µL of FA. Next, individual samples were cleaned up on Sep-Pak™ C-18 cartridges (Waters Corp., Milford, MA, USA) and dried in a Vacufuge™ vacuum centrifuge concentrator (Eppendorf AG, Hamburg, Germany). The residues were stored at −80 °C until proteomics-based analyses.

### 2.4. Nanoflow Liquid Chromatography-Coupled Tandem Mass Spectrometry Using Data-Dependent and Data-Independent Acquisitions (DDA and DIA nLC-MS/MS)

Vacuum-dried peptides were reconstituted in 5% (*v*/*v*) aqueous ACN containing 0.1% (*v*/*v*) FA to a concentration equivalent to 1 μg/μL total protein. Data were collected in both DDA and DIA modes on a Thermo Exploris 480 mass spectrometer equipped with an Easy-Spray™ source, operated in positive polarity mode and coupled to a Vanquish^TM^ Neo LC system (all from Thermo Fisher Scientific, San Jose, CA, USA). For both acquisition modes, 2 μL of reconstituted sample was separated by reverse-phase chromatography on an Acclaim PepMap Neo C18 column (150 mm × 75 μm i.d., 2 μm particle size, 100 Å pore size; Thermo Fisher Scientific) at a flow rate of 300 nL/min, using 0.1% FA in water (Solvent A) and 0.1% FA in 80/20 (*v*/*v*) ACN/water (Solvent B).

DDA acquisition used a 90 min gradient (1% to 28% B), followed by a 10 min column wash at 99% B. Full MS-ddMS2 parameters: S-lens radiofrequency (RF) level, 50; MS1 scan range, 375–1475 *m*/*z*; full MS normalized automatic gain control (AGC) target, 3 × 10^6^; maximum injection time (IT), 50 ms; Orbitrap resolution, 120,000 (at *m*/*z* 200), acquired in profile mode with an AGC target of 400,000; data-dependent MS2 scans targeted precursor ions with charge ≥2, using a 1.6 *m*/*z* isolation window, higher-energy collisional dissociation (HCD), normalized collision energy, (NCE) of 30, acquired in centroid mode.

DIA acquisition used a 60 min gradient (5% to 25% B over 40 min, then 25% to 65% B over 20 min), followed by a 10 min column wash at 99% B, adapted from a previously described protocol [39]. MS1 parameters: scan range, 390–1010 *m*/*z*; Orbitrap resolution, 60,000; maximum IT, auto; normalized AGC target, 100% (1 × 10^6^), acquired in centroid mode. MS2 acquisition used an 8 *m*/*z* variable isolation window scheme, Orbitrap resolution of 15,000, AGC target of 100%, loop count of 75, default charge state (z) of 3, and HCD fragmentation (NCE 27%), acquired in centroid mode.

### 2.5. Data Analysis

All searches relied on the same UniProtKB *Rattus norvegicus* protein database (species: *Rattus norvegicus*, 2025; 36,206 entries). Raw data files were processed assuming trypsin as the digestion enzyme, with a maximum of one missed cleavage and carbamidomethylation of cysteine as a fixed modification. Peptide lengths of 6–25 residues and peptide charges (z) ranging from 2 to 5 were selected [31]. A false discovery rate (FDR) of 1% was applied across all platforms unless otherwise stated. For DDA-based discovery-driven proteomics, database searches were performed using Scaffold (version 5.3.3) and Scaffold DDA (version 6.5.0) from Proteome Software (Portland, OR, USA), with their built-in MSFragger, maintaining a minimum protein probability of 99% by ProteinProphet and peptide probability of 95% by PeptideProphet, with at least two peptides per protein required for identification. Label-free quantification (LFQ) was performed using spectral counting; Scaffold’s integrated QRILC module (Quantile Regression Imputation of Left-Censored data) was utilized to handle missing values prior to statistical testing. Statistical analysis was conducted using the software’s built-in module. DEPs were identified using unpaired *t*-tests with Benjamini–Hochberg (BH) correction for multiple testing, with the significance set at *p* < 0.05. DIA data were analyzed using the AI-powered search algorithm CHIMERYS™ (MSAID GmbH, Garching, Germany), incorporating deep learning-based fragmentation prediction (inferys_4.7.0) and integrated within Proteome Discoverer (version 3.2). Fragment mass tolerance was set at 20 ppm and target FDR at 1%. Protein quantification was performed using the MS2 Apex method of the software, with the significance assessed through Proteome Discoverer’s internal algorithm relying on unpaired *t*-tests (*p* < 0.05) with BH correction for multiple testing. Principal component analysis (PCA) and volcano plots were generated in Scaffold (version 6.5.0). For the second set of experiments dedicated to the quantitative survey of selected protein markers from the first experimental design, raw data files generated from the Naïve, DHED, and Vehicle groups were processed using Scaffold DIA (version 4.3.1, Proteome Software, Portland, OR, USA) in conjunction with ProteoWizard (version 3.0.19254). Raw files were converted to mzML format, followed by retention time alignment and spectral library searching against a Rat_prosit_generated_library.dlib, with peptide and fragment mass tolerances of 20 ppm and 10 ppm, respectively. Search criteria included trypsin digestion with a maximum of one missed cleavage, carbamidomethylation of cysteine as a fixed modification, and peptides with charge states of 2 to 5 and lengths of 6 to 30 amino acid residues. Peptide identification was filtered using Scaffold DIA’s integrated Percolator (version 3.0.1). Quantification was performed using the software’s implementation of EncyclopeDIA (version 5.4.17), selecting ≥ 2–3 peptides and the five highest-quality fragment ions per peptide [31]. Statistical differences in protein abundance between groups (DEPs) were assessed using ANOVA followed by unpaired *t*-tests (*p* < 0.05) with BH correction, implemented through Scaffold DIA’s built-in statistics module, and fold change cutoffs ≥1.5 or ≤−1.5.

### 2.6. Bioinformatics

Proteins showing statistically significant differences in expression (DEPs) from the complementary DDA and DIA analyses were combined into a master list by merging proteins common to both platforms with those uniquely identified by either DDA or DIA. Proteins showing opposite regulation between the two platforms were excluded from the master list. This master list was then submitted to IPA^®^ (QIAGEN Inc., Redwood City, CA, USA) to investigate patterns of regulation through the associated canonical pathways, diseases, and functional networks, as well as protein interaction networks. Statistical significance was determined using IPA^®^’s built-in right-tailed Fisher’s exact test. Visualization and network analyses were performed using several IPA^®^ modules. Bubble chart was generated by IPA^®^. The platform’s Pathway Explorer (PE) module was used to build protein interaction networks incorporating receptors as well as disease and function categories. To highlight specific segments within complex networks, the Focus View feature of IPA^®^ was utilized to fade distant nodes. The Molecule Activity Predictor (MAP) tool was also used to predict the functional activation or inhibition of specific signaling pathways, diseases and functions categories, based on overall z-scores, which assign directionality to a function or pathway with activation and inhibition color-coded in orange and blue, respectively [31].

## 3. Results

### 3.1. ORX-Mediated Proteome Alterations in the Male Rat VC Revealed by Complementary DDA and DIA nLC-MS/MS

**Figure 1:**
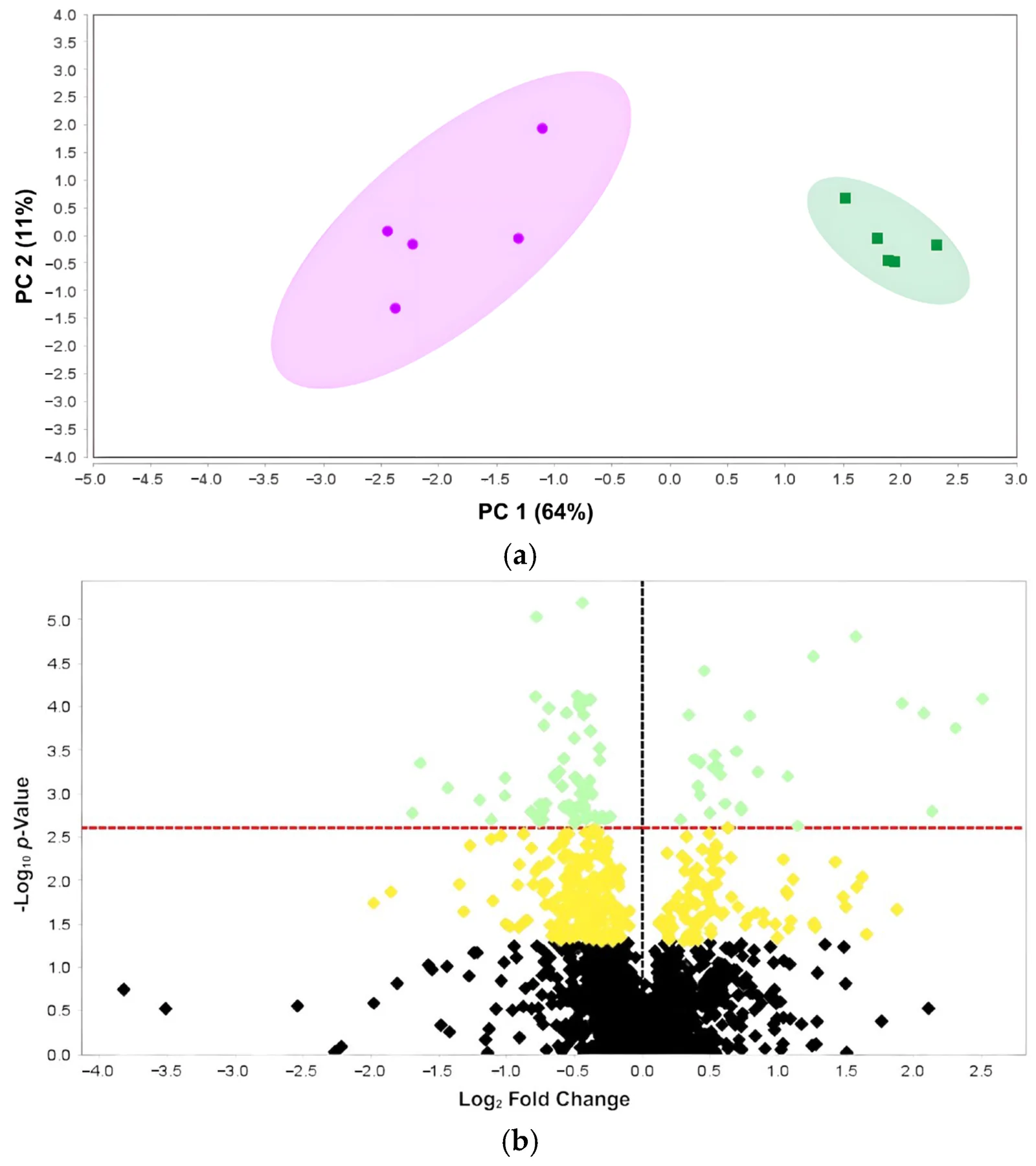
Scaffold-based proteomic analyses of the male BN rat VC. (**a**) PCA plot illustrates the separation between the VC proteome of the naïve (intact, green) and ORX (magenta) groups. (**b**) Volcano plot depicting DEPs between the naïve (intact) and ORX groups. Green—significant after BH multiple testing correction; yellow—significant before BH correction; black—not significant. Left panel represents downregulated proteins, while the right panel represents upregulated proteins.

We identified with high confidence over 3500 proteins at 1% FDR. To maximize the proteome coverage [40], DEPs from both DDA and DIA platforms were integrated into a master list. More than 500 proteins were found to be significantly regulated between the naive (intact) and ORX-VC (Table S1), of which 198 proteins were downregulated and 312 were upregulated following ORX. PCA and volcano plots are shown in Figure 1 to visually summarize these findings. For biological context, IPA^®^’s curated Knowledge Base linked these DEPs to more than 50 disease- and physiological function-related categories enriched in neurological functions (Table S2), and over 200 canonical pathways (Table S3). Additionally, 22 protein interaction networks were constructed from the submitted master list (Table S4). Notably, OS emerged as a predominant and recurring theme across multiple enriched categories, pathways, and networks, positioning it as a central mechanistic driver of ORX-induced neurological dysfunction.

**Figure 2:**
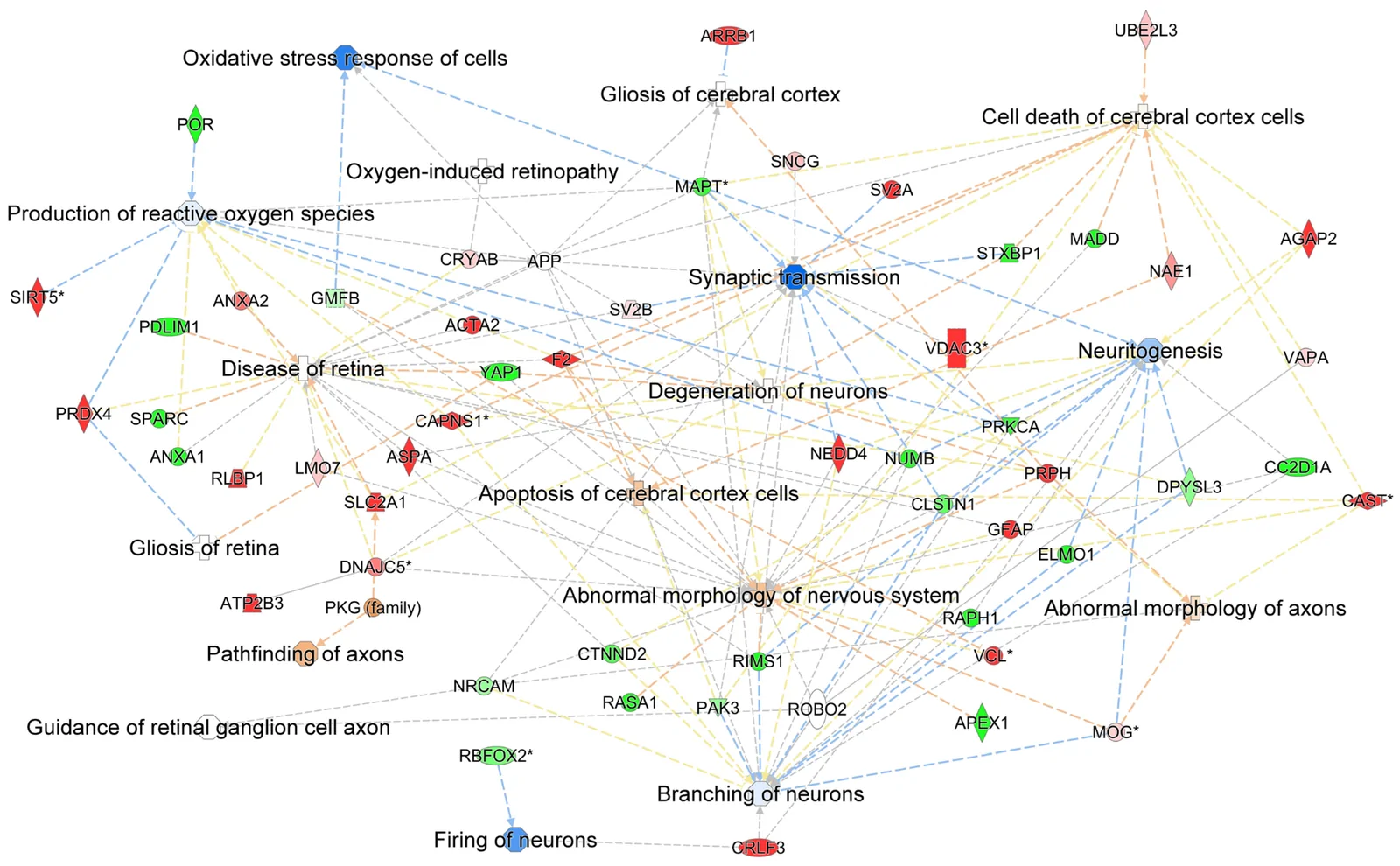
IPA^®^-generated network of ORX-induced proteome alterations associated with OS and neurodegenerative functions in the male BN rat VC. The network was constructed based on DEPs identified between naïve (intact) and ORX animals, with IPA^®^ predicting activation or inhibition of disease- and function-related networks. Line annotations: orange solid/dashed—predicted activation; blue solid/dashed—predicted inhibition; yellow dashed—effect cannot be predicted; grey solid/dashed—direct/indirect relationship without predicted directionality. Node color reflects the direction and magnitude of change: red indicates upregulation, and green indicates downregulation in the dataset, with shade intensity corresponding to the magnitude of change in protein expression. Red and green reflect actual measurements, while orange and blue reflect IPA^®^-predicted activation/inhibition. Edge lines represent known protein–protein interactions according to IPA^®^’s Knowledge Base. The abbreviated protein names are listed in Table S5a, * denotes multiple isoforms identified by IPA^®^.

Using IPA^®^’s MAP tool, we overlaid retina-associated enrichment annotations from IPA^®^’s curated Knowledge Base onto our VC-derived DEPs, building on our prior proteomic characterization of the ORX retina [31] and the established anatomical connection between retina and VC [17]. These annotations shown in Figure 2 do not represent measurements from retinal tissue in the present study; rather, they reflect shared molecular pathway terms within IPA^®^’s database that are linked to our VC dataset. This approach allowed us to map IPA^®^-predicted activation and inhibition of biological functions, illustrating a pattern of possible degenerative involvement in both the retina and the VC.

This network shows links between representative DEPs and IPA^®^-curated annotations of OS-related ocular neuropathology, applied here to our VC-derived dataset. These include cellular OS responses such as ROS production (e.g., NADPH–cytochrome P450 reductase, POR), oxygen-induced retinopathy (e.g., alpha-crystallin B chain, CRYAB), impaired RGC axon guidance and pathfinding, abnormal axon morphology, dysregulated neuronal firing and branching, progressive neuronal degeneration, reduced synaptic transmission, and impaired neurogenesis (e.g., myelin oligodendrocyte glycoprotein, MOG). IPA^®^ also linked several DEPs to retinal functions. These links reflect shared molecular pathways in IPA^®^’s Knowledge Base, not direct retinal data from this study. Together, these predicted associations point to a possible extension of ORX-induced effects (gliosis, abnormal nervous system morphology, and apoptotic cell death) across the visual pathway. This remains a hypothesis based on shared IPA^®^ annotations, not directly tested by comparing retinal and VC tissues within this study.

**Figure 3:**
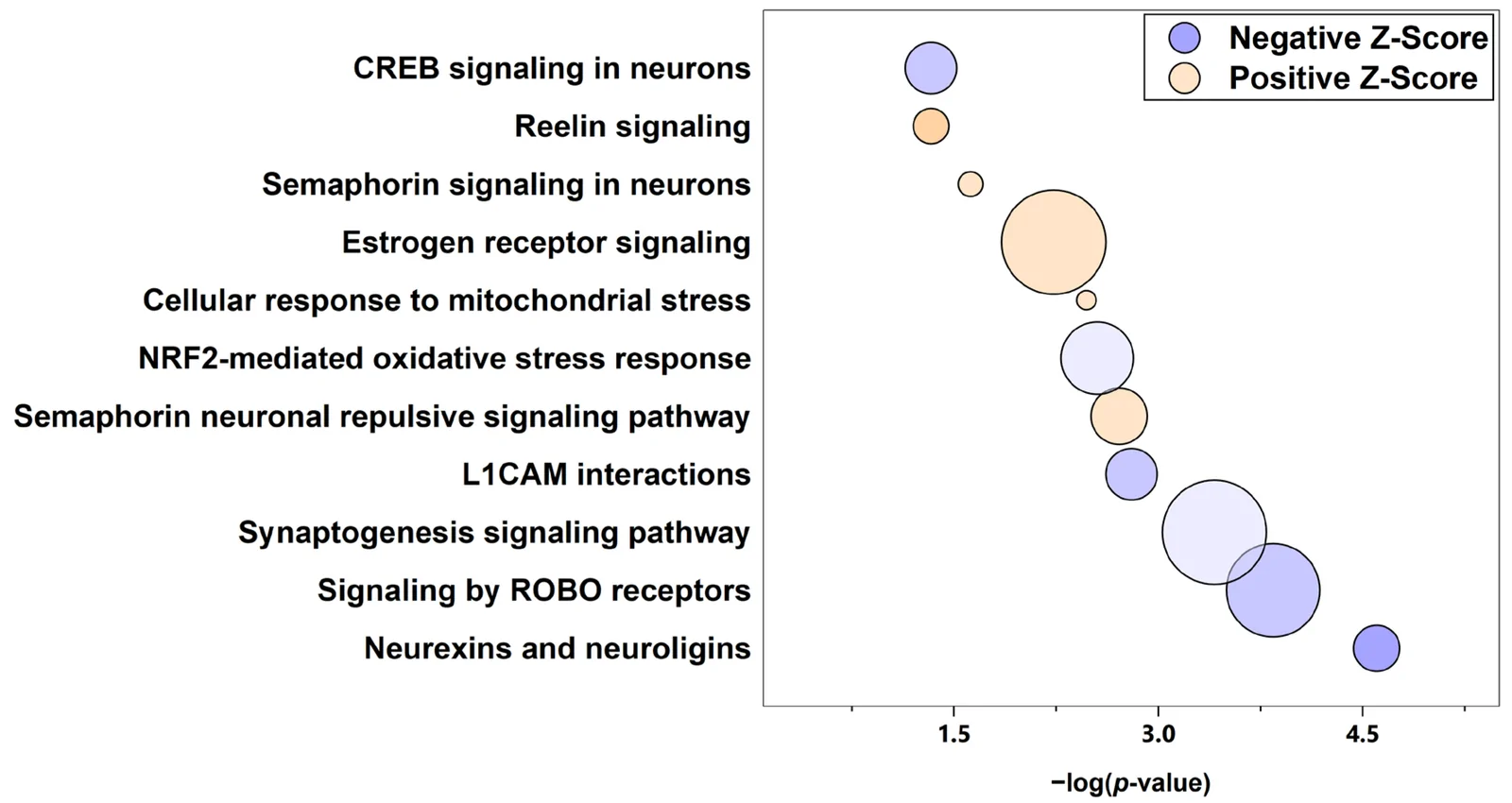
ORX-regulated proteins involved in top canonical pathways associated with the OS response in the male BN rat VC. Bubble size indicates the number of proteins overlapping with each pathway. Orange and blue colors represent predicted activation and inhibition, respectively.

Figure 3 presents a bubble chart generated by IPA^®^ from our measured DEPs (Table S1); these canonical pathways are directly or indirectly implicated in OS response, including the involvement of nuclear factor erythroid 2-related factor 2 (NRF2), ESR, and cellular response to mitochondrial stress. Consistent with our previously reported findings in ORX-retina [31], ESR signaling was also among the top pathways in ORX-VC. Figure 3 also shows downregulation of cyclic AMP response element-binding protein (CREB) signaling in neurons. CREB signaling is thought to help balance neurodegeneration and neuroprotection under OS [41].

Based on IPA^®^’s Knowledge Base, Figure 3 highlights several pathways annotated as involved in retina-to-VC neurosensory relay, including cascades tied to neurogenesis and to DEPs identified in the VC following ORX, among them ROBO receptor and reelin activity, the semaphorin neuronal repulsive pathway, L1CAM interactions, and synaptogenesis. Notably, our analysis also revealed inhibition of the neurexin–neuroligin signaling cascades, implicating trans-synaptic degenerative events in the ORX VC. Neurexins are located on the presynaptic membrane and neuroligins on the postsynaptic membrane; these well-known trans-synaptic cell adhesion proteins interact across the synaptic cleft to mediate bidirectional communication and are essential for synapse organization, stabilization, and functional integrity [42].

**Figure 4:**
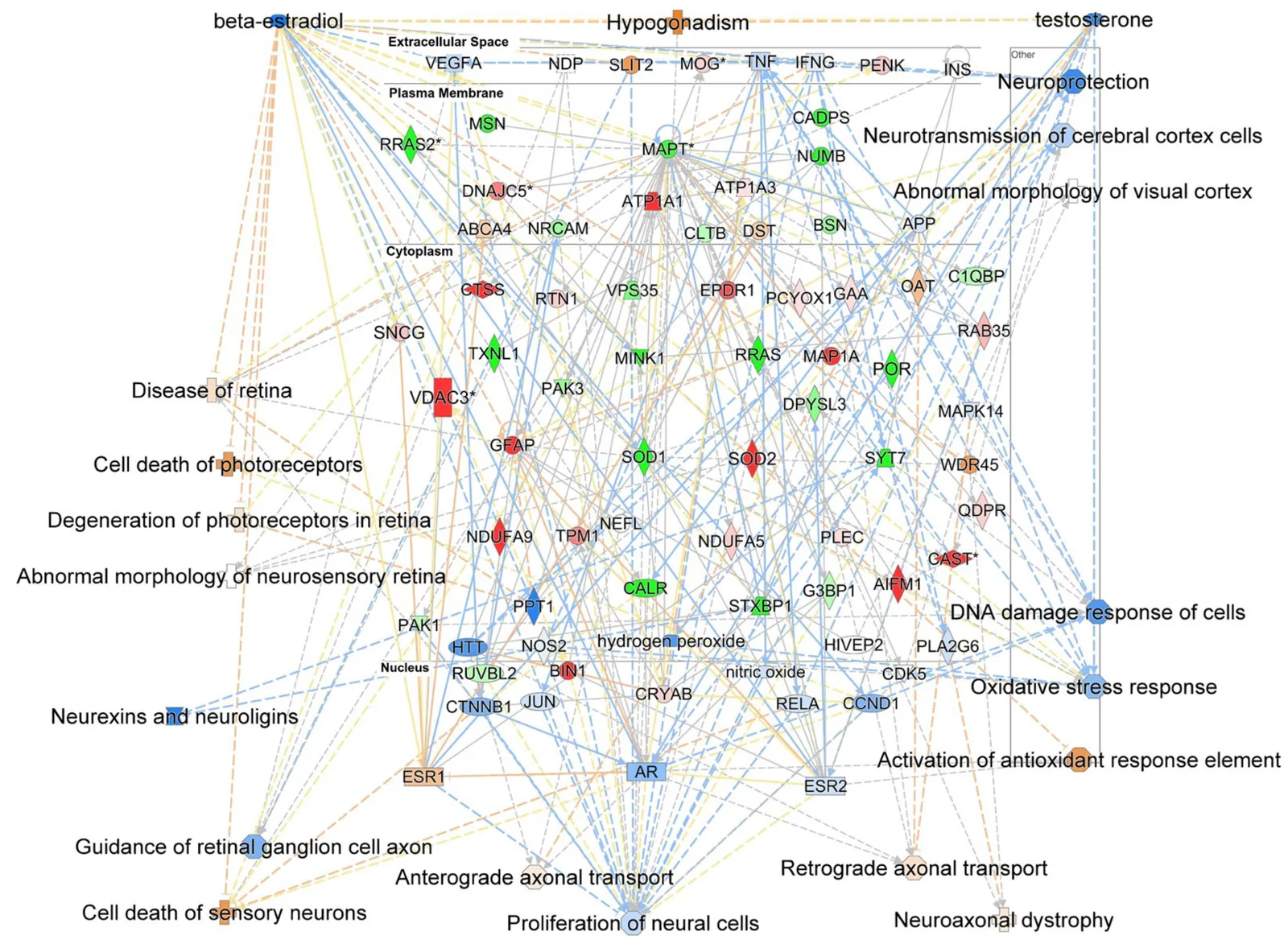
Interaction network generated by IPA^®^’s PE among the ORX-impacted DEPs in the VC (SOD2, VDAC3, CRYAB, GFAP, SNCG, BSN, SYT7, DPYSL3, POR, and MOG), incorporating E2, T, and their respective nuclear receptors (ESR1, ESR2, and AR), as well as components of the OS response and neurodegenerative pathways. Line annotations: blue solid/dashed line—inhibition/decrease; orange dashed line—activation/increase; yellow dashed line—effect cannot be predicted; grey solid/dashed line—direct or indirect relationship, respectively, without predicted directionality. Node color: red—upregulated; green—downregulated; shade intensity reflects the magnitude of change in protein expression. Protein name abbreviations are listed in Table S5b, * denotes multiple isoforms identified by IPA^®^.

By integrating the hypothesis-generating PE tool of IPA^®^ into the protein interaction network associated with neurological function and disease (Table S4), we constructed a potential roadmap toward ORX-induced neurodegeneration in the VC (Figure 4). Similar to our previous findings in the ORX BN male retina [31], AR signaling did not reach the significance threshold among the top canonical pathways associated with the OS response in the VC (Figure 3). However, given its crucial involvement in male hormonal regulation [43], we further investigated the DEPs identified in the VC upon male sex hormone deprivation by ORX, mirroring our previous approach applied to the ORX retina [31]. Accordingly, our analysis included T, E2, their nuclear receptors (AR, ESR1, ESR2), and OS-related functions IPA^®^ annotated as part of retina-to-VC signaling, to map how these factors relate to neurodegeneration in this context.

Figure 4 shows the regulatory network built by combining IPA^®^’s machine learning tools with other heuristic tools provided by the software’s PE tool. This network maps out pathways that may lead to neurodegeneration in the VC under hypogonadism, when both T and E2 are lost following ORX. The network further shows E2 directly activating (orange dashed line) antioxidant response elements (AREs), while no such link was found for T, similar to what we found in the ORX-retina [31].

Figure 4 further shows the connection of AR to synaptotagmin-7 (SYT7) and glial fibrillary acidic protein (GFAP), while ESR1 and ESR2 modulate γ-synuclein (SNCG), voltage-dependent anion channel 3 (VDAC3), SYT7, superoxide dismutase 2 (SOD2), GFAP, and dihydropyrimidinase-related protein 3 (DPYSL3). This network suggests that the loss of T is potentially associated with the dysregulation of POR and SOD2 in the ORX-VC, while E2 was predicted to directly regulate GFAP, SNCG, and SOD2, suggesting SOD2 may be regulated by both T and E2 pathways. In addition, the antioxidant response element (ARE) was implicated as being regulated not only by E2 but also by ESRs.

To build a roadmap linking sex hormone loss (ORX), the retina, and the VC, we overlaid the network with IPA^®^-predicted diseases and functions, along with their activation or inhibition states. The left side of Figure 4 shows retinal functions annotated by IPA^®^’s Knowledge Base, including increased retinal disease, cell death, photoreceptor degeneration, and abnormal retinal morphology, based on shared molecular associations with our ORX-VC DEPs, not direct measurement of retinal tissue in this study. We also mapped our DEPs to hallmark neurosensory events linked to vision loss [42,44]: inhibition of neurexin and neuroligin signaling across the synapse, loss of RGC axon guidance, death of sensory neurons, neuroaxonal dystrophy in optic nerves, and reduced neural cell proliferation. In contrast, both anterograde and retrograde transport were predicted to increase after ORX, suggesting a compensatory response to degeneration. IPA^®^ also predicted a decrease in DNA damage and OS response, suggesting some cellular defense pathways stay partly active. Overall, these predicted changes point to a loss of neuronal function, marked by abnormal morphology in ORX-VC and reduced neurotransmission in cortical cells, as shown on the right side of Figure 4.

### 3.2. Quantitative Survey of E2-Driven Attenuation of ORX-Induced Disruption in a Selected Panel of the Male Rat VC Proteins Following DHED Eye Drop Treatments

**Figure 5:**
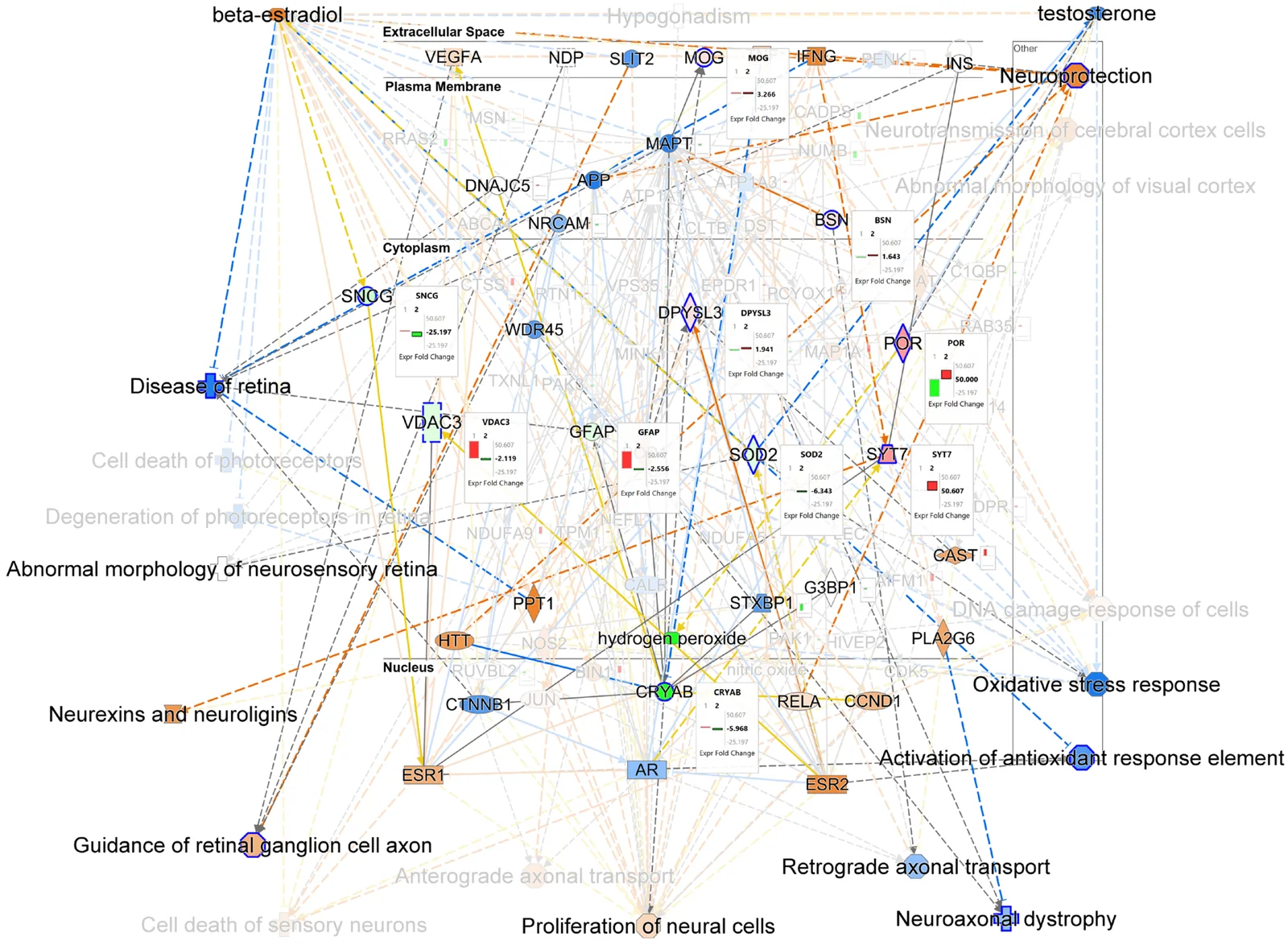
Focus view of the interaction network shown in Figure 4, generated by IPA^®^’s PE among the selected DEPs (SOD2, VDAC3, CRYAB, GFAP, SNCG, BSN, SYT7, DPYSL3, POR, and MOG), E2, T, and their respective nuclear receptors (ESRs and AR), and key elements of OS and neuroprotective response pathways, with quantitative proteomic data (with and without DHED treatment) overlaid via IPA^®^’s Data Overlay platform. Line annotations: orange—activation/increase; blue—inhibition/decrease; yellow dashed—effect cannot be predicted; grey—direct or indirect relationship without predicted directionality (solid: direct; dashed: indirect relationship). Node color: red—upregulation; green—downregulation; shade intensity reflects the magnitude of change in protein expression. Inset: protein expression for the ORX group (1st bar) and DHED-treated group (2nd bar); the naïve group is not shown, as it serves as the fold-change baseline. Abbreviations are listed in Table S5b.

In Figure 5, we show a focused view of the network displayed in Figure 4, generated by fading distant nodes and highlighting only the panel of selected ORX-impacted DEPs shown in Figure 4. To assess the proteome’s response to DHED treatment (i.e., E2 replacement), this network was further integrated with quantitative proteomic data obtained without (first bar, Figure 5 inset) and with (second bar, Figure 5 inset) DHED administration, enabling direct visualization of the IPA^®^-predicted E2-mediated biological impact at the network level. This integrated analysis implicates a reduction in disease burden within the IPA^®^-annotated Retinal Disease category, potentially reflecting molecular pathways shared with the VC, alongside a coordinated upregulation of key neurobiological processes, including enhanced expression of the trans-synaptic adhesion molecules neurexins and neuroligins, improved RGC axon guidance, and increased neural cell proliferation. Additionally, IPA^®^’s curated Knowledge Base predicted downregulation of OS-prone molecular switches represented by AREs [31], suggesting DHED treatment reduced the OS burden. Altogether, these network-level observations imply a shift toward enhanced neurotransmission in cerebral cortical cells, warranting functional validation of the potential neuroprotective benefits of DHED-derived E2.

**Figure 6:**
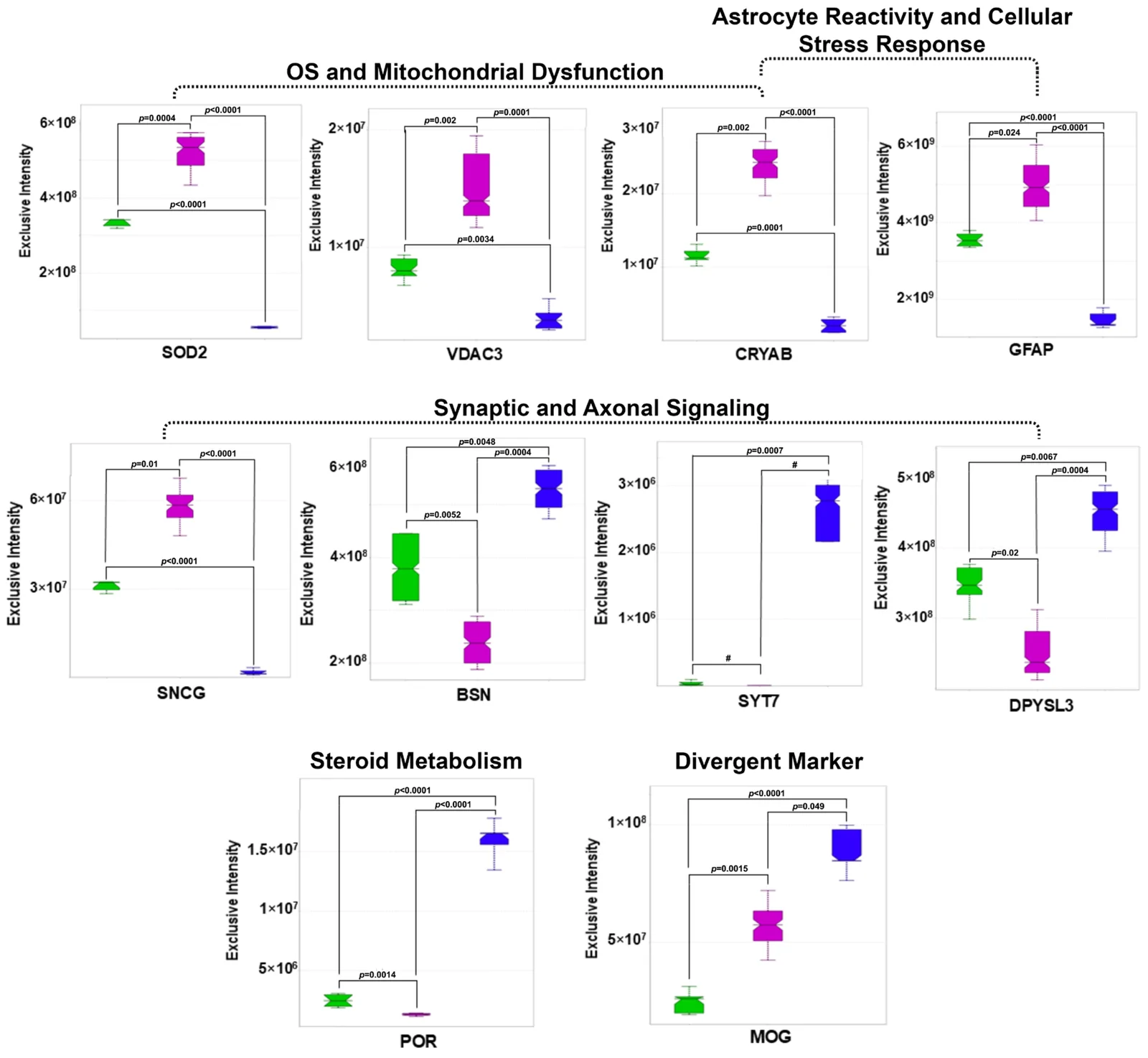
Scaffold DIA-based quantitative survey of key DEPs from Figure 4 and Figure 5, predicted by IPA^®^ to be involved in neuroprotection. Box plots: green—naïve (intact); magenta—vehicle; blue—DHED. Protein descriptions and transitions used for quantitation are in Tables S6a and S6b, respectively; raw intensities for all samples are listed in Table S6c. One-way ANOVA with post hoc Tukey–Kramer tests (*n* = 5/group) showed statistically significant differences between all group pairs for all listed proteins; *p*-values are displayed on the chart; #—not tested).

Figure 6 illustrates the effects of ORX and DHED treatment on this protein panel (listed in Table S6a with LFQ transitions in Table S6b). The effects of ORX (magenta boxes) and DHED treatment (blue boxes) are shown relative to the naïve group (green boxes).

These quantitative assessments show that ORX-induced proteome dysregulation in the VC can be beneficially modulated by central E2 supplementation, despite the loss of gonadal T and its estrogenic downstream metabolites following ORX. Altogether, the proteins in this panel represent, as seen in Table 1, several functional categories: OS and mitochondrial dysfunction (SOD2, VDAC3, CRYAB), astrocyte reactivity and cellular stress response (CRYAB, GFAP), synaptic and axonal signaling (SNCG, BSN, SYT7, DPYSL3), and steroid metabolism (POR). MOG stands apart as a divergent marker.

## 4. Discussion

The present study pursued two objectives: establishing a discovery-driven proteomic dataset of the ORX-VC and testing whether DHED (10β,17β-dihydroxyestra-1,4-dien-3-one) eye drop-mediated E2 delivery, without reestablishing circulating testosterone, would be sufficient to attenuate ORX-induced cortical proteome disruption. Consistent with our previous studies involving ocular neurodegeneration, DHED eye drops were used [11,20], and the rapid formation of E2 from this ophthalmic dosage form has been previously demonstrated [59].

This route preferentially produces high DHED-derived E2 concentrations in the retina, owing to the superior transcorneal flux of DHED achieved with our dosage form [59], whereas systemic DHED administration would favor broader brain-wide E2 formation, including within the VC, without peripheral hormonal exposure in either case [36,38,60]. Cortical E2 concentration was not measured here, as this discovery-driven hypothesis-generating study was designed to explore whether DHED eye drop treatment alone, thus without reestablishing circulating testosterone, is sufficient to attenuate ORX-induced VC proteomic disruption in a selected panel of key proteins, rather than to resolve the complex underlying retina-cortex signaling mechanism. As the retina and VC form a functionally coupled system linked by retinogeniculate and corticofugal pathways, the relative contribution of retina-derived versus direct cortical E2 action to the observed and predicted VC changes was not addressed in this exclusively MS-based proteomic-focused study, which represents the logical continuation of our previously published work on ORX-induced changes in the corresponding retina [31].

Using a dual-platform DDA/DIA strategy [40,61] with multiple complementary search algorithms (Scaffold, Scaffold-DDA and CHIMERYS) and strict validation criteria, we identified over 3500 high-confidence proteins and 500 DEPs between naïve (intact) and ORX groups (Figure 1, Table S1), with a 30% overlap between the two platforms. IPA^®^ predicted these DEPs to be mainly linked to OS, mitochondrial dysfunction, neuroinflammation, and impaired synaptic transmission (Figure 2 and Figure 4, Tables S2–S4). Although our dataset was derived exclusively from VC tissue, IPA^®^ analysis also annotated several DEPs to retinal functions based on enrichment against IPA^®^’s curated Knowledge Base, rather than direct retinal measurements; while this does not demonstrate propagation across the visual pathway, it is consistent with the hypothesis that ORX-induced proteomic disruption is not strictly retina-specific. Neurological and Ophthalmic Disease categories were significantly overrepresented (69 and 25 proteins, respectively; Table S2), with predicted links to retinal neuron disease, sensory neuron death, and disrupted axonal transport and synaptic transmission.

Throughout the present study, IPA^®^-predicted pathway associations are used to interpret our findings; these reflect plausible mechanistic links drawn from IPA^®^’s curated Knowledge Base and do not represent direct functional validation of these associations.

The VC retains a high degree of neuroplasticity well into adulthood in both humans and rodents [62]. IPA^®^ associated several predicted dysregulated pathways with neuronal communication and synaptic function (Figure 2 and Figure 3, Tables S2 and S3). Synaptic transmission was predicted to be downregulated, suggesting impaired synaptic activity. Along with the predicted activation of cortical cell apoptosis and reduced neurogenesis, this DEP pattern is consistent with a neurodegenerative state at the protein-annotation level (Figure 2). Ocular-related diseases were also linked to these DEPs, including oxygen-induced retinopathy, retinal gliosis, and retinal disease, implying some overlap in disease-related proteins between the retina and VC, consistent with our prior retinal findings (Figure 2, Table S2) [31]. Several pathways and proteins involved in synaptic maintenance were predicted to be downregulated, including neurexins and neuroligins as well as ROBO receptors and L1CAM (Figure 3 and Figure 4, Table S3). Notably, ESR signaling was among the top canonical pathways (Figure 3), while AR signaling did not meet the significance cut-off, consistent with our earlier findings in the male BN rat ORX-retina model [31].

Within the visual system, the retina experiences particularly high OS levels due to its high metabolic demand and constant light exposure [63]. Consequently, dysregulation of retinal antioxidant systems may represent a vulnerability that could plausibly contribute to neurodegeneration extending beyond the eye, though this has rarely been studied outside the retina. A study reported downstream atrophy in the lateral geniculate nucleus and VC [64], and one rodent glaucoma model found elevated ROS and reduced mitochondrial ATP synthesis in the VC [65]. While we did not directly examine the retina here, we previously found that ORX BN rats showed an IPA^®^-predicted increase in retinal OS susceptibility [31], consistent with the OS-related dysregulation, mitochondrial stress, and NRF2-mediated response predicted here in the VC (Figure 2, Figure 3 and Figure 4, Tables S2 and S3). NRF2 is a master regulator of antioxidant activity [66].

SOD2 and cytoplasmic superoxide dismutase 1 (SOD1) expression in the ORX-VC showed an inverse relationship: SOD1 was downregulated, and SOD2 was upregulated (Figure 4, Figure 5 and Figure 6, Table 1), consistent with our previous findings in the ORX retina [31]. Elevated mitochondrial ROS may drive SOD2 upregulation, potentially indicating reduced mitochondrial efficiency and increased protein/DNA oxidation [67,68]. SOD2 was similarly elevated in the VC of glaucomatous rodents, alongside impaired mitochondrial function and elevated ROS, as measured by Western blot [65]. This inverse relationship warrants further study, since SOD1 or SOD2 upregulation can lead to different outcomes in neurological disease [45,69]. With DHED treatment, SOD2 was downregulated, falling below naïve levels (Figure 6, Table 1). The literature is mixed on how sex hormones regulate SOD2, likely due to tissue-specific effects. In rat cardiomyocytes exposed to flutamide, an AR inhibitor, both mRNA and protein expression rose, while ORX alone only modestly raised SOD2, suggesting AR (via dihydrotestosterone) may suppress SOD2 [70]. While E2 has also been shown to reduce SOD2 mRNA, this does not necessarily reflect activity [71,72].

Another ORX-affected protein, VDAC3, is a mitochondrial porin involved in OS management, mitochondrial function, and biogenesis [46]. This protein has also been linked to male fertility through its roles in spermatogenesis and sperm motility [73]. Transgenic knockout models highlight its regulatory role, and treatment with ROS-inducing agents suggests it may play a compensatory role under OS [74]. VDAC3 expression was elevated in the ORX group (Figure 2, Figure 4, Figure 5 and Figure 6, Table 1), possibly reflecting ROS-driven upregulation, though its functional impact on mitochondrial efficiency was not directly assessed here. With DHED treatment, VDAC3 was downregulated below naïve levels (Figure 6, Table 1). Little is known about VDAC3 in relation to sex hormones; to our knowledge, this may be the first study to identify it as sex hormone-responsive, and further work on its functional role is warranted.

Crystallins are heat-shock proteins best known for maintaining lens translucency, but they also serve neuroprotective roles across several tissues, including neurons [47]. Like SOD2, CRYAB was upregulated in the ORX group (Figure 2, Figure 4, Figure 5 and Figure 6, Table 1). CRYAB has been shown to modulate OS pathways and inhibits apoptosis [48], in part through cysteine residues that scavenge ROS. Given IPA^®^’s predicted activation of OS-related pathways (Figure 2), the rise in CRYAB may reflect a compensatory response to neuronal stress, consistent with reports of CRYAB elevation in trauma-injured brain [75]. Cellular stressors like OS and protein aggregation may drive CRYAB expression as a defense mechanism, which may also explain its reported link to amyloid beta [49]. With DHED treatment, CRYAB was downregulated (Figure 6, Table 1), potentially reflecting IPA^®^-predicted attenuation of the ORX-induced stress response by DHED-derived E2 (Figure 2, Figure 4, Figure 5 and Figure 6, Table 1).

CRYAB is commonly associated with glial fibrillary acidic protein (GFAP), a widely used reactive astrocyte marker [49,50] that was also upregulated in the ORX group (Figure 2, Figure 4, Figure 5 and Figure 6, Table 1). In the VC, GFAP is mainly expressed by astrocytes, and is tied to aging and neurodegenerative conditions, including astrogliosis [49,75]. CRYAB also directly stabilizes GFAP during trauma, such as traumatic brain injury [75]. Elevated GFAP has been linked to reactive astrocytes, neurological disease, glial scarring, and increased ROS [76]. Its relationship with sex hormones is relatively well established: GFAP is expressed not only in astrocytes but also in androgen-producing Leydig cells in males, and both T and E2 are known to regulate its expression [77]. With DHED treatment, GFAP expression was similarly downregulated (Figure 6, Table 1). Given this reduction alongside IPA^®^’s predicted upregulation of neuroprotection-related pathways (Figure 5), this decrease may point toward reduced astrocyte activation, potentially consistent with the suppression of reactive astrogliosis in the VC driven by E2 replenishment. However, as our study assessed protein expression rather than histological or functional readouts, direct measurement of GFAP-positive cell density will be needed to confirm whether glial scarring or astrogliosis is affected. This is consistent with prior reports linking sex hormone deprivation to accelerated brain-aging via elevated GFAP [76].

Synuclein proteins maintain and regulate neuronal communication and synaptic function. Dysfunction of their expression or activity is generally tied to protein aggregation, which contributes to neurodegenerative diseases such as Parkinson’s disease [51]. Most SNCG research has focused on cancer and the peripheral nervous system, but growing evidence now points to a role in CNS neurodegeneration [51,78]. Elevated expression of SNCG has been reported following insults, such as exposure to ROS-inducing agents like 6-hydroxydopamine, which increases SNCG in both the prefrontal cortex and the hippocampus [79]. Overexpression and oxidation of SNCG induce synucleinopathy, with aggregate and fibril formations that lead to severe neurodegeneration and reduced lifespan in transgenic mouse models [78,80]. SNCG is not inherently pathological, but pathological aggregation is thought to occur once a threshold is crossed. We found elevated SNCG in the VC of the ORX group (Figure 2, Figure 4, Figure 5 and Figure 6, Table 1). This finding, combined with the IPA^®^-predicted dysregulation of axonal transport, neuronal cell death, and OS pathways, may point to increased vulnerability to neurodegeneration (Figure 2 and Figure 4). With DHED treatment, SNCG was downregulated (Figure 6, Table 1), consistent with our earlier finding that DHED treatment reduced elevated SNCG in the glaucomatous rat retina [11], suggesting that SNCG is tied to ORX-related stress pathways and responsive to E2.

The release of synaptic vesicles at the synapse is a key regulatory point for neuronal communication, especially in sensory circuits [81]. Consistent with IPA^®^-predicted dysregulation of neuronal communication in the VC after ORX (Figure 4), BSN, a presynaptic scaffold protein located within the synaptic terminal active zone [52,82], was downregulated in the ORX group (Figure 4, Figure 5 and Figure 6, Table 1). Along the visual pathway, BSN is expressed from the retina to the VC, and its loss causes major vision loss in transgenic animals [83]. Its role in neurodegenerative diseases is not straightforward; patients with multiple system atrophy show elevated BSN mRNA levels, while BSN knockdown protected against tauopathy in an Alzheimer’s mouse model [52,84,85]. Other studies indicate a role for BSN in presynaptic vesicle release and autophagy, with its suppression causing structural changes resembling degenerating neurons [86]. With DHED treatment, BSN expression rose close to naïve levels (Figure 6, Table 1). BSN may be E2-regulated: one study using immunocytochemistry found that E2 increased the dendritic spine number and reversed synapse loss in a schizophrenia model, though it remains unclear whether these spines overlapped with presynaptic BSN protein, making the BSN–E2 link indirect [87]. To date, however, no proteomics evidence directly relates BSN to E2. Given BSN’s many interactions, its altered expression likely has complex effects and may relate to several neurodegenerative diseases; more work is needed to clarify its role in synaptic maintenance.

Another synapse-associated protein, synaptotagmin-7 (SYT7), is involved in synaptic plasticity, synaptic vesicle exocytosis, and calcium sensing [53]. Suppression of SYT7 mRNA has been reported in the neurodegenerative APP/PS1 Alzheimer’s mouse model through a transcriptomic study [88]; in that model, restoring SYT7 expression reduced inflammation and improved cognitive performance. While its role in the VC has not been specifically assessed, to our knowledge, we are the first to report that ORX leads to a significant decrease in SYT7 expression in the VC (Figure 4, Figure 5 and Figure 6, Table 1). With DHED treatment, SYT7 expression was significantly increased (Figure 6, Table 1). These changes may affect synaptic plasticity and communication, which future studies should directly assess.

DPYSL3 (also called CRMP4) belongs to a family of proteins important for brain development and regulates multiple neuronal pathways [54,55,89]. We found reduced DPYSL3 in the ORX group (Figure 2, Figure 4, Figure 5 and Figure 6, Table 1), which may weaken its neuroregulatory functions and contribute to the neuronal deficits predicted by IPA^®^, though this functional interpretation was not directly tested. Elevated DPYSL3 has been linked to axonal growth and guidance, regeneration, synapse formation, and vesicle recycling in spinal, hippocampal, and retinal neurons [55,89,90,91], and it has also been identified as a cancer target, where it promotes tumor growth and metastasis [92]. With DHED treatment, DPYSL3 rose (Figure 6, Table 1), which IPA^®^ associated with resistance to neurodegeneration-related pathways. Since DPYSL3 is also regulated by phosphorylation (e.g., by GSK3β) [93], expression levels alone may not capture its full functional state; phosphoproteomic analysis would help clarify this in future work.

POR is a cytochrome (CYP) P450 oxidoreductase found mainly in the liver but also in the brain, where it helps metabolize xenobiotics and neurosteroids [56,57]. It forms complexes with numerous CYP enzymes, mediating electron transfer from NADPH to CYP. Brain CYP expression is diverse, with glia and neurons expressing different subtypes [57]. The VC lacks detailed CYP characterization, but it expresses CYP19A1 (aromatase, which converts T to E2), while other cortical regions express CYP2B6, CYP1A1, and CYP1A2, all of which depend on POR [94,95]. CYP expression was unchanged after ORX (Table S1), but POR was found downregulated in the ORX group (Figure 2, Figure 4, Figure 5 and Figure 6, Table 1), possibly pointing to reduced CYP function without altered CYP expression, as reported before [96]. Although enzymatic activity was not directly measured here, this could suggest less efficient CYP activity after ORX and a potential vulnerability to toxins and xenobiotics. This may be particularly relevant for cancer patients on androgen deprivation therapy (ADT), where chemotherapeutics like docetaxel have been linked to “chemobrain,” an accelerated cognitive decline attributed to OS and cellular toxicity [97,98]. ADT’s impact on visual function and pathway integrity, however, remains virtually unexplored. Our findings suggest ADT may induce a neurodegenerative proteomic signature in the VC (Figure 2 and Figure 4, Tables S2 and S3), consistent with our prior findings in the ORX retina [31], raising the possibility that ADT-treated patients face heightened risk of visual pathway neurodegeneration and vision impairment. In turn, with DHED treatment, POR expression rose sharply (Figure 6, Table 1). Since POR donates electrons to CYP enzymes [94], this may reflect increased CYP activity driven by DHED-derived E2, pointing to potentially increased steroid metabolism. Direct measurement of POR/CYP enzymatic activity and downstream steroid metabolites will be necessary to test this hypothesis.

While constituting a relatively minor component of the myelin sheath, MOG is embedded in the outermost leaflet of the myelin sheath surrounding neuronal axons [58]. In oligodendrocytes, MOG serves as a marker of maturation and may have functional roles of its own [58]. MOG peptides are commonly used to trigger autoimmunity and model diseases like multiple sclerosis in animals, but MOG’s physiological role remains poorly understood [58,99,100]. Exposing cultured oligodendrocytes to anti-MOG antibodies disrupts their cytoskeleton, causing microtubule retraction and actin disorganization, suggesting MOG may help regulate oligodendrocyte activity and myelination [101,102]. MOG expression was upregulated in the ORX group (Figure 2, Figure 4, Figure 5 and Figure 6, Table 1); since its cysteine residues are oxidation-sensitive and have previously served as an injury signal in retinal neurons [103], measuring MOG’s cysteine oxidation directly could be a better OS indicator than expression alone. Unlike every other marker in this panel, MOG did not trend back toward naïve (intact) levels with DHED, and instead rose even further (Figure 6, Table 1). T is known to regulate myelination and remyelination, via AR signaling or aromatization to E2 [104], but no prior study has linked sex hormones to MOG expression. IPA^®^’s Knowledge Base shows no direct interaction between MOG and E2, T, or their receptors at the protein level; any link appears to be noncanonical, likely mediated through microtubule-associated protein tau (MAPT), as shown in Figure 4. To our knowledge, this is the first report connecting E2 and ORX to MOG expression; its unusual pattern raises the possibility of a distinct currently uncharacterized regulatory pathway.

## 5. Conclusions

This study provides the first evidence that androgen deprivation, through bilateral orchiectomy, is associated with proteome disruption in the VC. This is consistent with our prior findings in the ORX retina [31] and raises the possibility that patients undergoing ADT face a heightened risk for visual pathway dysfunction. Quantitative proteomic assessment of a selected protein panel (Figure 6, Table 1) showed that, independently of circulating testosterone, DHED eye drop-mediated E2 delivery to the retina shifted the expression of these ORX-dysregulated cortical proteins toward levels observed in naïve (intact) controls, apart from MOG. IPA^®^ pathway analysis further predicted this shift to be associated, in part, with reduced OS signaling and altered axon guidance-related activity. Among the assessed proteins, we identified several E2-responsive candidates in the VC, including MOG, whose regulation by E2 has not, to our knowledge, been previously described. These findings support our treatment strategy to mitigate androgen deprivation-induced dysregulation of these key proteins in the VC, independent of testosterone, and warrant broader proteome-level and functional validation of its potential benefits.

## Figures and Tables

**Table 1:** List of key protein markers in Figure 6 with fold changes and core biological functions. Negative fold change—downregulation; positive fold change—upregulation. Fold changes were calculated from abundance averages reported in Table S6c.

Protein ID	Fold Change			Core Function
	Impact of ORX	Impact of DHED		
	ORX/Naive	DHED/ORX	DHED/Naive	
SOD2	2	−11	−6	Mitochondrial antioxidant defense [45].
VDAC3	3	−5	−2	Mitochondrial porin involved in OS management and biogenesis [46].
CRYAB	2	−12	−5	Molecular chaperone scavenges ROS, inhibits apoptosis [47,48].
GFAP	2	−4	−2	Reactive astrocyte marker linked to aging, gliosis, and neurodegeneration [49,50].
SNCG	2	−37	−20	Neuronal communication and synaptic maintenance [51].
BSN	−2	2	1.4	Presynaptic scaffold protein at the active zone [52].
SYT7	L ^1^	H ^1^	23 ^2^	Synaptic vesicle exocytosis and calcium sensing [53].
DPYSL3	−2	2	1.3	Axonal growth, guidance, and synapse formation [54,55].
POR	−2	11	6	Mediates electron transfer to CYPs, involved in steroid and xenobiotic metabolism [56,57].
MOG	2	2	4	Myelin component with a maturation-marker role [58]; divergent marker

^1^ L and H denote lower and higher, respectively—not calculated because abundances in three out of five ORX samples decreased below threshold set by Scaffold DIA. ^2^ Including two imputed values by Scaffold DIA for the naïve group.

## Data Availability

The mass spectrometry proteomics data have been deposited to the ProteomeXchange Consortium via the PRIDE [105] partner repository with the dataset identifiers PXD080391(DOI: 10.6019/PXD080391) and PXD080450.

## Supplementary Materials

The following supporting information can be downloaded at https://www.mdpi.com/article/10.3390/antiox15080976/s1, Table S1: DEPs comparing the VC of ORX and naïve groups. Proteins that met the fold change cutoffs ≥1.5 or ≤−1.5 and were statistically significant based on *t*-test with BH correction; Table S2: Disease, molecular, and functional pathways generated by IPA^®^ based on the submitted DEPs comparing the VC of ORX and naïve groups; Table S3: IPA^®^-generated canonical pathways related to OS, based on the submitted DEPs comparing the VC from ORX and naïve groups; Table S4: List of all protein interaction networks generated from the DEPs between the ORX and naïve groups; Table S5: (a) List of protein abbreviations and overlaid functions shown in Figure 2; (b) List of protein abbreviations and overlaid functions shown in Figure 4; Table S6: (a) Scaffold DIA-based quantitative proteomics involving the selected panel of protein markers (Figure 6, Table 1) using the group comparison mode for native endogenous proteotypic peptides; (b) Transitions used for quantitative assessment of the selected protein panel shown in Figure 6 and Table 1. (c) Abundances used in Scaffold DIA-based quantitative proteomics involving the selected panel of protein markers (Figure 6 and Table 1) using the group comparison mode for native endogenous proteotypic peptides.

## Abbreviations

The following abbreviations are used in this manuscript:

ABC	Ammonium bicarbonate
ACN	Acetonitrile
ADT	Androgen deprivation therapy
AGC	Automatic gain control
ANOVA	Analysis of variance
APP/PS1	Amyloid precursor protein/Presenilin1
AR	Androgen receptor
ARE	Antioxidant response element
BH	Benjamini–Hochberg
BN	Brown Norway
BSN	Bassoon
CRYAB	Alpha-crystallin B chain
CNS	Central nervous system
CREB	Cyclic AMP response element-binding protein
CRMP	Collapsin response mediator protein
CYP	Cytochrome P450
DDA	Data-dependent acquisition
DHED	10β,17β-dihydroxyestra-1,4-dien-3-one
DEP	Differentially expressed protein
DIA	Data independent acquisition
DPYSL	Dihydropyrimidinase-related protein
E2	17β-estradiol
ESR	Estrogen receptor
FA	Formic acid
FDR	False discovery rate
GFAP	Glial fibrillary acidic protein
GSK3β	Glycogen synthase kinase 3 beta
HCD	Higher-energy collisional dissociation
IOP	Intraocular pressure
IPA^®^	Ingenuity Pathway Analysis^®^
IT	Injection time
L1CAM	L1 cell adhesion molecule
LC	Liquid chromatography
LFQ	Label-free quantitation
MAP	Molecule activity predictor
MAPT	Microtubule-associated protein tau
MOG	Myelin–oligodendrocyte glycoprotein
MS	Mass spectrometry
MS/MS	Tandem mass spectrometry
NADPH	Nicotinamide adenine dinucleotide phosphate
NCE	Normalized collision energy
nLC	Nanoflow liquid chromatography
NRF2	Nuclear factor erythroid 2-related factor 2
ON	Optic nerve
ORX	Orchiectomy
OS	Oxidative stress
PCA	Principal component analysis
PE	Pathway explorer
POR	NADPH-cytochrome P450 reductase
RF	Radiofrequency
ROBO	Roundabout receptor
ROS	Reactive oxygen species
RGC	Retinal ganglion cell
SOD	Superoxide dismutase
SYT7	Synaptotagmin-7
T	Testosterone
VC	Visual cortex
VDAC3	Voltage-dependent anion channel 3
